# Genome-Wide Identification, Characterization, and Expression Analysis of NF-Y Gene Family in *Ginkgo biloba* Seedlings and *GbNF-YA6* Involved in Heat-Stress Response and Tolerance

**DOI:** 10.3390/ijms241512284

**Published:** 2023-07-31

**Authors:** Tongfei Wang, Helin Zou, Shixiong Ren, Biao Jin, Zhaogeng Lu

**Affiliations:** College of Horticulture and Landscape, Yangzhou University, Yangzhou 225009, China; mx120120794@stu.yzu.edu.cn (T.W.); mx120220806@stu.yzu.edu.cn (H.Z.); dx120220142@stu.yzu.edu.cn (S.R.)

**Keywords:** *Ginkgo biloba*, nuclear factor Y, heat response, thermotolerance

## Abstract

Nuclear factor Y (NF-Y) transcription factors play an essential role in regulating plant growth, development, and stress responses. Despite extensive research on the NF-Y gene family across various species, the knowledge regarding the NF-Y family in *Ginkgo biloba* remains unknown. In this study, we identified a total of 25 NF-Y genes (seven GbNF-YAs, 12 GbNF-YBs, and six GbNF-YCs) in the *G. biloba* genome. We characterized the gene structure, conserved motifs, multiple sequence alignments, and phylogenetic relationships with other species (Populus and Arabidopsis). Additionally, we conducted a synteny analysis, which revealed the occurrence of segment duplicated *NF-YA*s and *NF-YB*s. The promoters of *GbNF-Y* genes contained cis-acting elements related to stress response, and miRNA–mRNA analysis showed that some *GbNF-YA*s with stress-related cis-elements could be targeted by the conserved miRNA169. The expression of *GbNF-YA* genes responded to drought, salt, and heat treatments, with *GbNF-YA6* showing significant upregulation under heat and drought stress. Subcellular localization indicated that GbNF-YA6 was located in both the nucleus and the membrane. Overexpressing *GbNF-YA6* in ginkgo callus significantly induced the expression of heat-shock factors (GbHSFs), and overexpressing *GbNF-YA6* in transgenic Arabidopsis enhanced its heat tolerance. Additionally, Y2H assays demonstrated that GbNF-YA6 could interact with GbHSP at the protein level. Overall, our findings offer novel insights into the role of *GbNF-YA* in enhancing abiotic stress tolerance and warrant further functional research of *GbNF-Y* genes.

## 1. Introduction

Nuclear factor Y (NF-Y) is a family of heterotrimeric transcription factors that exists in nearly all eukaryotes, including yeast, mammals, and plants. The NF-Y family is known to consist of the CBF domain (CCAAT-binding factor) and HAP domain (heme activator protein). On the basis of sequence differences, NF-Y family is generally classified into three highly conserved subfamilies: NF-YA (HAP2 or CBF-B), NF-YB (HAP3 or CBF-A), and NF-YC (HAP5 or CBF-C) [1]. As a heterotrimeric transcription factor, NF-YB and NF-YC interactions produce a dimer complex in the cytoplasm, which then combines with NF-YA to complete the assembly of the heterotrimeric complex in the nucleus [2]. Unlike animals and yeast, in which each subfamily within *NF-Y* has a single gene [3], higher plants have 10 or more genes for each subfamily that can form different NF-Y trimes. For example, *Arabidopsis thaliana* and *Oryza sativa* contain 36 (10 NF-YAs, 13 NF-YBs, and 13 NF-YCs) and 34 NF-Y genes, respectively [4,5]. The NF-Y complex can specifically bind to CCAAT elements to regulate the expression of their target genes [6], while approximately 7–8% of plant genes contain active CCAAT-boxes in their promoters. Collectively, the abundance of *NF-Y*s, combinatorial diversity (NF-Y trimers), and extensive binding sites across the plant genome indicate that *NF-Y*s may have substantial potential for regulatory capacity in the plant development process and stress response. Indeed, *NF-Y* genes have been found to play vital roles in the resistance to abiotic stress (especially in drought) in many plants. For instance, *AtNF-YB1* can improve drought resistance in Arabidopsis, and an orthologous NF-Y factor (*ZmNF-YB2*) in maize was proven to have an equivalent activity [7]. Moreover, *TaNF-YB3* and *OsNF-YA7* enhance the drought tolerance of crops by regulating the ABA-associated signaling pathway [8,9]. In addition, NF-Ys have been found in response to salt [10,11], cold [12], UV-B [13], nutrient stress [14], osmotic stress [14], and oxidative stress [13].

*Ginkgo biloba* is one of the oldest relic gymnosperms, known as a “living fossil”. It is grown globally as a landscape tree and also utilized as a medicinal plant, owing to its rich secondary metabolites. *G. biloba* has experienced climate and geological changes in its long evolutionary history and survived, indicating its strong tolerance to environmental stresses. Many studies have focused on the tolerance of *G. biloba*. With the release of the ginkgo genome sequence, more gene families associated with resistance traits in *G. biloba* are being revealed [15,16]. However, information on the NF-Y family in *G. biloba* remains scarce.

In this work, we totally identified 25 *GbNF-Y*s in the *G. biloba* genome and characterized their phylogenetic tree, conserved motifs, gene structures, and responsive expressions to heat, drought, and salt, respectively. On the basis of these expression profiles, we screened an important GbNF-YA6 gene that dramatically responds to both heat and drought. Furthermore, we identified the interactive proteins of GbNF-YA6 using a yeast two-hybrid assay and validated its function via transgentic ginkgo calli and Arabidopsis plants. Our results provide a valuable reference for the future study of the mechanism and function of the NF-Y family in *G. biloba* and other species.

## 2. Results

### 2.1. Identification and Analysis of the NF-Y Family in G. biloba

**Figure 1:**
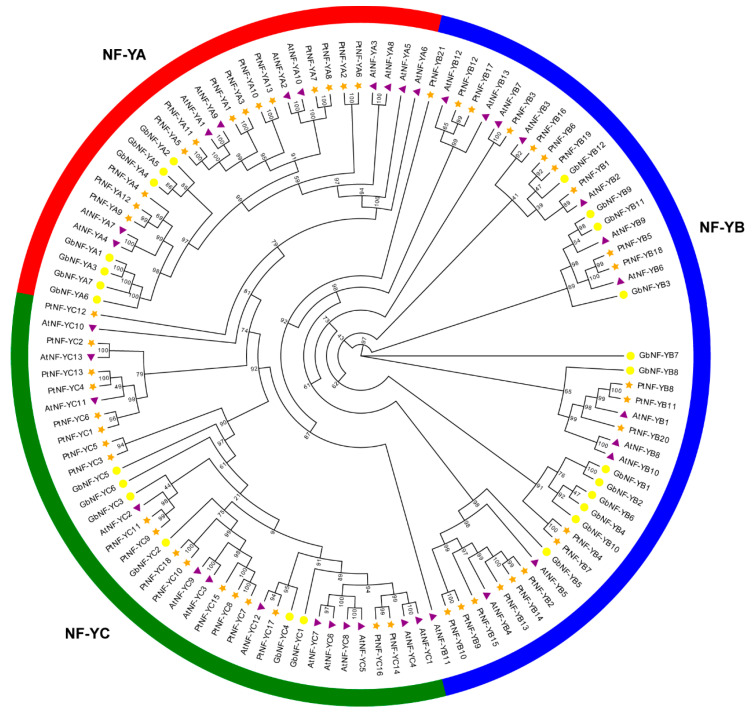
Phylogenetic relationships among NF-Y proteins in Ginkgo, Arabidopsis, and Populus. The phylogenetic tree was constructed using IQ-TREE (v.1.6.9) via the maximum likelihood method with bootstrap values for 1000 replicates. Different colors were assigned to represent distinct subfamilies; red segments in the circles indicate NF-YA subfamilies, blue segments in the circles indicate NF-YB subfamilies, and green segments in the circles indicate NF-YC subfamilies.

Through multiple sequence searches and domain confirmation, a total of 25 nonredundant members of the NF-Y family were identified from the *G. biloba* genome. On the basis of their encoded subunits, the ginkgo NF-Y family could be divided into three subfamilies: (1) the NF-YA subfamily consisting of seven genes, which were named *GbNF-YA1* to *GbNF-YA7* according to their order in chromosomal position; 12 members of the NF-YB subfamily, which were named *GbNF-YB1* to *GbNF-YB12*; six genes belonging to the NF-YC subfamily, which were named *GbNF-YC1* to *GbNF-YC*6. Moreover, we performed the phylogenetic analysis of NF-Y members from Ginkgo, Arabidopsis, and Populus using 113 NF-Y protein sequences (Figure 1, Supplementary File S1). The phylogenetic tree showed that 113 NF-Y members could also be divided into three main groups distinguished by three different colors (red, green, and blue), which were consistent with the classifications of the GbNF-Ys subfamily. In each group, we found some homologous genes (such as *GbNF-YB9*, *GbNF-YB11*, *AtNF-YB9*, *PtNF-YB5*, *GbNF-YB18*, *AtNF-YB6*, and *GbNF-YB3*) from Ginkgo, Arabidopsis, and Populus. This suggests that these paired genes may share similar biological functions.

The proteins encoded by the *GbNF-YA* genes varied in length, ranging from 182 (*GbNF-YA7*) to 406 (*GbNF-YA2*) amino acids (aa) in ORF length. The calculated molecular weight was between 20.2 (*GbNF-YA7*) and 45.5 kDa (*GbNF-YA2*), and their calculated isoelectric point (PI) ranged from 7.07 (*GbNF-YA4*) to 9.61 (*GbNF-YA7*). In the GbNF-YB subfamily, their length of amino acids (aa), molecular weight (kDa), and pI ranged from 510 (GbNF-YB8) to 906 (*GbNF-YB11*), 41.2 (*GbNF-YB8*) to 73.8 (*GbNF-YB11*), and 5.08 (*GbNF-YB2*) to 5.23 (*GbNF-YB9*), respectively. The corresponding ranges of GbNF-YC subfamily members were 193 (*GbNF-YC6*) to 276 aa (*GbNF-YC1*) in ORF length, 21.9 (*GbNF-YC6*) to 31.2 kDa (*GbNF-YC1*) in molecular weight, and 5.72 (*GbNF-YC2*) to 7.77 (*GbNF-YC5*) in pI. The distributions of these variables are shown in Table S1.

### 2.2. Motif and Structural Analyses of GbNF-Y Genes

**Figure 2:**
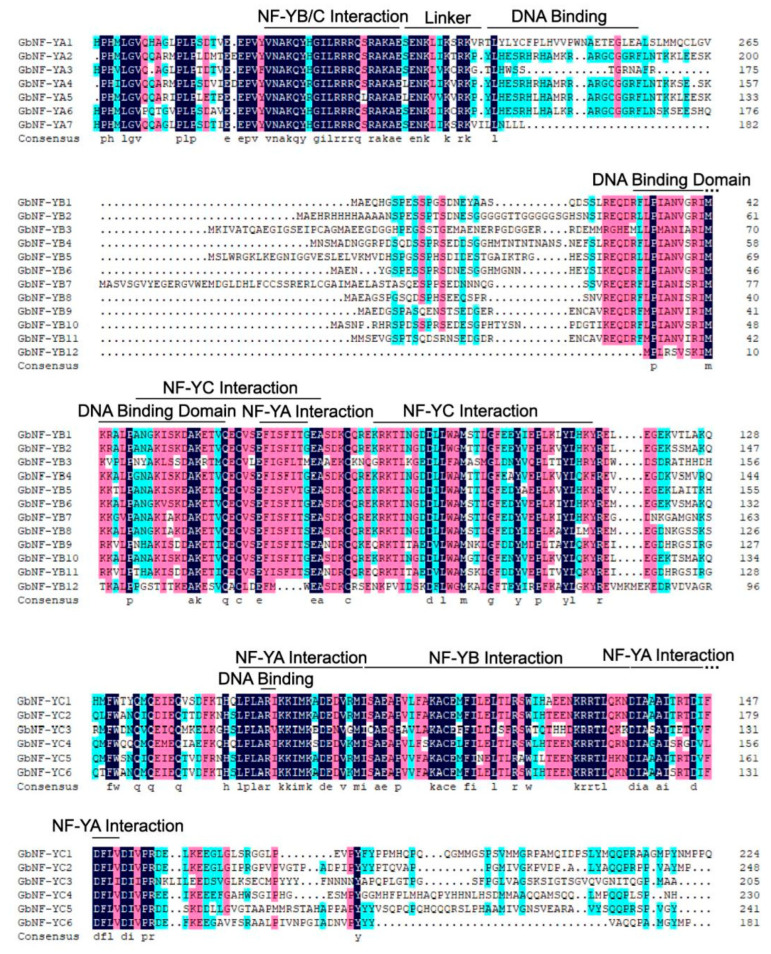
Ginkgo NF-Y family alignment. The core, conserved domains of 25 GbNF-Y proteins were compared and visualized using DNAMAN (URL: http://www.bio-soft.net accessed on 22 July 2023).

Multiple sequence alignments showed that GbNF-Y proteins possess specific conserved regions and relatively variable N-terminal or C-terminal transcriptional regulation domains. On the basis of the results of the sequence alignment (Figure 2), the GbNF-YA conserved region has two subdomains: a domain for NF-YB/NF-YC interaction and a domain for DNA-binding domain. The GbNF-YB contains the NF-YA and NF-YC interaction domains and a DNA-binding domain. Similarly, the GbNF-YC included three conserved subdomains: the NF-YA interaction domain, the NF-YB interaction domain, and the DNA-binding domain.

**Figure 3:**
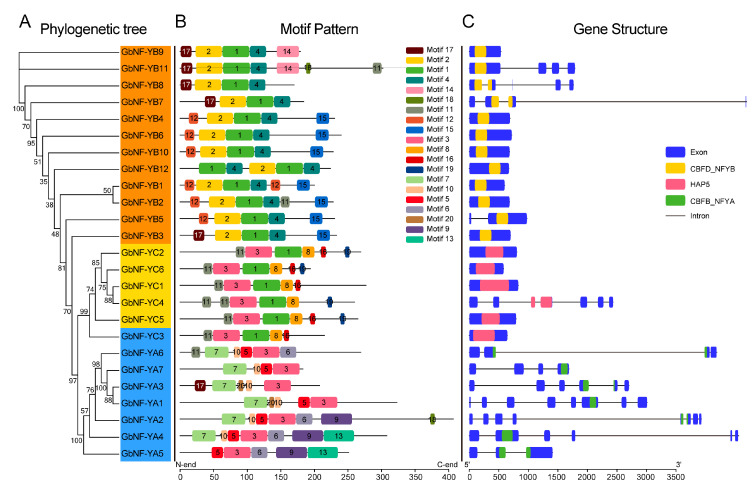
The phylogeny, conserved motifs, and gene structures of 25 *GbNF-Y* genes. To construct phylogenetic trees, we used the neighbor-joining method implemented by MEGA software and calculated bootstrap scores from 1000 replicates. The different subfamilies, NF-YA, -B, and -C, are indicated by blue, orange, and yellow, respectively. The GbNF-Y protein motifs were identified using the online MEME program, and different colored boxes represent different motifs, with their corresponding numbers in the center of the boxes. Furthermore, the blue rectangles and thin lines indicate the exons and introns, respectively, with some conserved domains shown in different colors.

On the basis of the alignment of the full-length amino-acid sequences, the phylogenetic tree including all *GbNF-Y* members was constructed as shown in Figure 3A. We found three paralogous pairs (*GbNF-YA1*/*GbNF-YA3*, *GbNF-YB1*/*GbNF-YB2*, and *GbNF-YC1*/*GbNF-YC4*) in the GbNF-Y family through analyzing the phylogenetic tree. To identify the putative motifs of the NF-Y family in *G. biloba*, the 20 conserved motifs of GbNF-Y family members were established as shown in Figure 3B using the MEME program (https://meme-suite.org/meme/ accessed on 22 July 2023). The results showed that the genes within the corresponding subfamily had high similarity, confirming that they belonged to the same gene subfamily. For example, motifs 1, 2, and 4 were present in the GbNF-YB subfamily, of which motif 2 annotated as histone-like transcription factor (CBFD_NFYB) only existed in the GbNF-YB. Similarly, motif 9, annotated as CCAAT-binding transcription factor (CBFB_NF-YA), was only displayed in the GbNF-YA subfamily. However, several conserved motifs (e.g., motif 1, 3) were shared among GbNF-YA, GbNF-YB, and GbNF-YC. 

Gene structure analysis of *GbNF-Y* members revealed structural diversity among different GbNF-Y subfamilies, as depicted in Figure 3C. In the GbNF-YB subfamily, the exon-intron structure of all members had a high similarity, except for *GbNF-YB7* and *GbNF-YB8*. Eight members (*GbNF-YB1*/*-2*/*-3*/*-4*/*-6*/*-9*/*-10*/*-12*) had one exon, while *GbNF-YB5-*/*-7*/*-8*/*-11* contained more than one exon. In contrast to the GbNF-YB and GbNF-YC subfamilies, we observed that members of the GbNF-YA subfamilies had more exons, ranging from three to six, with some intron intervals. In particular, the *GbNF-YA6*, *GbNF-YA2*, and *GbNF-YA4* gene structure comprised multiple small exons and overlong introns, which were not observed in the GbNF-YB and GbNF-YC subfamilies.

### 2.3. Chromosomal Location and Gene Duplication Analysis

**Figure 4:**
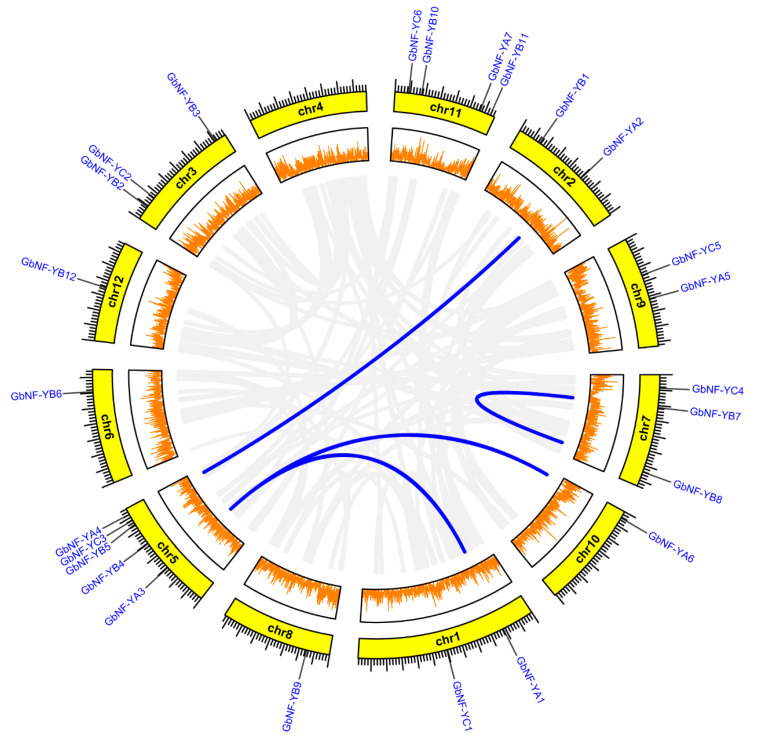
Localization and synteny of *GbNF-Y* genes in the *G. biloba* genome. The *GbNF-Y* genes were located on various chromosomes, with the exception of chromosome 4. The circles, arranged from outer to inner, represent *GbNF-Y* genes, chromosomes, gene density, and syntenic blocks, respectively. The blue lines indicate duplicated *GbNF-Y* genes.

According to the position information of the *GbNF-Y*s genes on the *G. biloba* chromosome, we found that the 25 genes were randomly distributed on 11 chromosomes (Figure 4). Notably, chromosome 5 had the largest number of genes (n = 5), followed by chromosome 11 (n = 4), chromosome 3, chromosome 7 (n = 3), chromosome 1 (n = 2), chromosome 9 (n = 2), chromosome 2 (n = 1), etc. Moreover, no gene was distributed on chromosome 4. Additionally, we did not observe any tandem duplication events within the GbNF-Y family. Instead, segment duplications occurred within and among the chromosomes. For instance, *GbNF-YB7* and *GbNF-YB8* were segment duplicates within chromosome 7, *GbNF-YA2* and *GbNF-YA4* were segment duplicates between chromosome 2 and 5.

### 2.4. cis-Acting Element Analysis of GbNF-Y Genes

**Figure 5:**
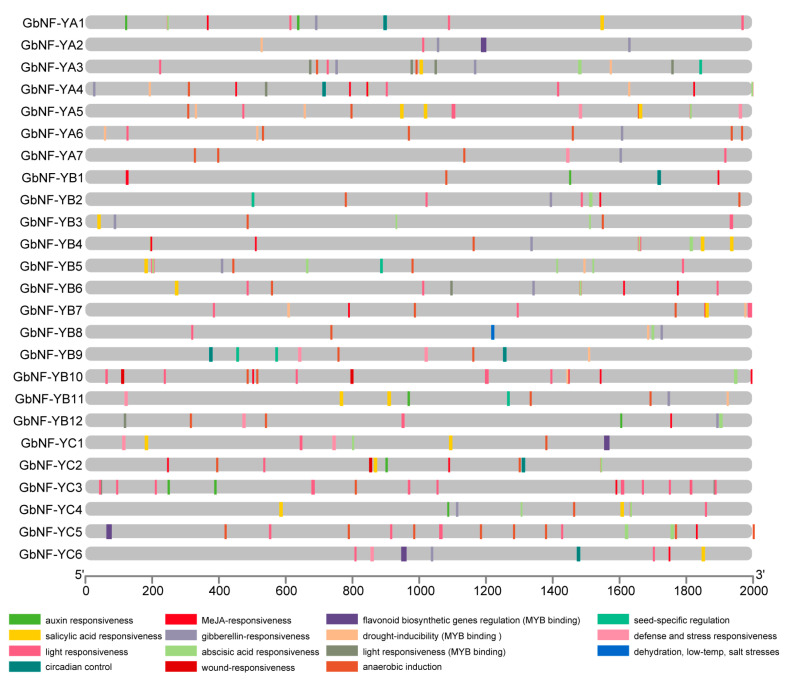
Distribution of cis-acting elements in the promoter sequences of the 25 GbNF-Y genes. Different cis-acting elements are colored by different colorants.

To demonstrate the possible biological function of *GbNF-Y* genes, we extracted the promoter regions (2 kb upstream of the transcription start site) of these genes for the identification of cis-regulatory elements. A number of cis-elements involved in responses to various abiotic stresses, growth development, and hormones were identified. Interestingly, light-responsive elements were present in the promoters of all *GbNF-Y* genes (Figure 5, Supplementary File S2). In addition, we found that MeJA-responsive elements were distributed in several *GbNF-Y* genes. For example, *GbNF-YA4* harbored four MeJA-responsive elements in its 2 kb upstream regulatory region. Moreover, auxin-responsive elements and flavonoid biosynthetic elements were rarely present in the sequences of the 2 kb upstream regulatory region. 

### 2.5. Comprehensive Analysis of microRNAs Targeting GbNF-Y Genes

**Figure 6:**
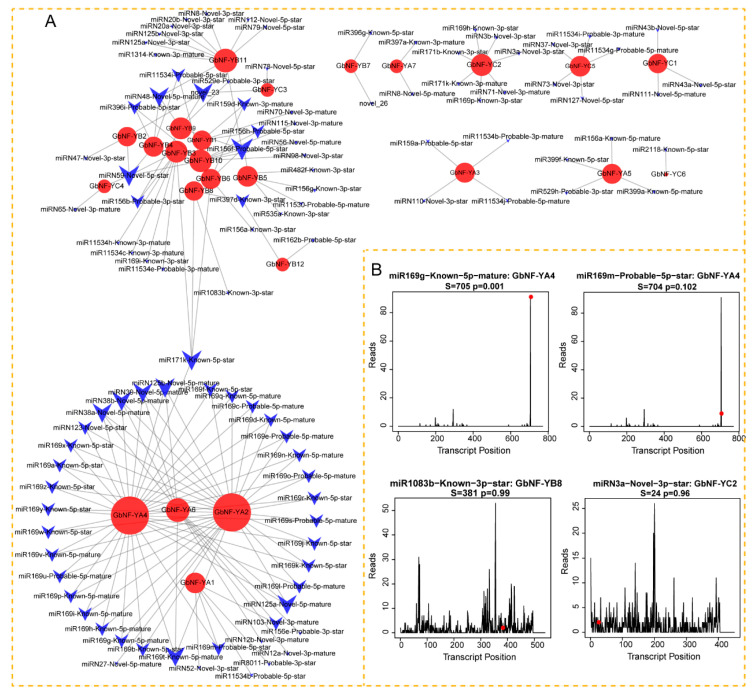
A schematic representation of targeted regulatory relations between putative miRNAs and their targets. (**A**) *GbNF-Y*s targeted by miRNAs were visualized by Cystoscape software. Blue arrows represent the miRNA, and red circles represent *GbNF-Y*s. The size of the red circles and arrows indicates the numbers. (**B**) *GbNF-Y*s were predicted to be cleaved by miRNAs on the basis of previous degradome data [17].

A total of 66 known miRNAs and 105 candidate novel miRNAs belonging to 47 miRNA families were used for target prediction. Our findings indicate that 105 miRNAs can target 24 *GbNF-Y*s (Figure 6A). To better understand microRNAs targeting *GbNF-Y* genes, we constructed a network to show the relationship between *GbNF-Y* genes and miRNAs (Figure 6A). Our results show that the miR169 family mainly targets the duplicated *GbNF-YA2* and *GbNF-YA4*, while other *GbNF-YB*s and *GbNF-YC*s are targeted by known or novel miRNAs. On the basis of our degradome data from our previous study [17], the miR169g-Known-5p-mature could cleave *GbNF-YA4* at a site 705 nucleotides from the five ends of mRNAs (*p* = 0.001). In addition, *GbNF-YA4* was also sliced at 704 nucleotides by miR169m-Probable-5p-star (Figure 6B, Supplementary File S3). The miR156 family was mainly targeting the GbNF-YB subfamily. *GbNF-YB8* was the target of miR1083b-Known-3p-star, and the site of *GbNF-YB8* was cleaved at 381 nucleotides. Moreover, the GbNF-YC subfamily was targeted by several novel miRNAs. MiRN3a-Novel-3p-star sliced *GbNF-YC2* at 24 nucleotides.

### 2.6. qRT-PCR Expression Profiles of GbNF-YA Genes under Different Treatments

**Figure 7:**
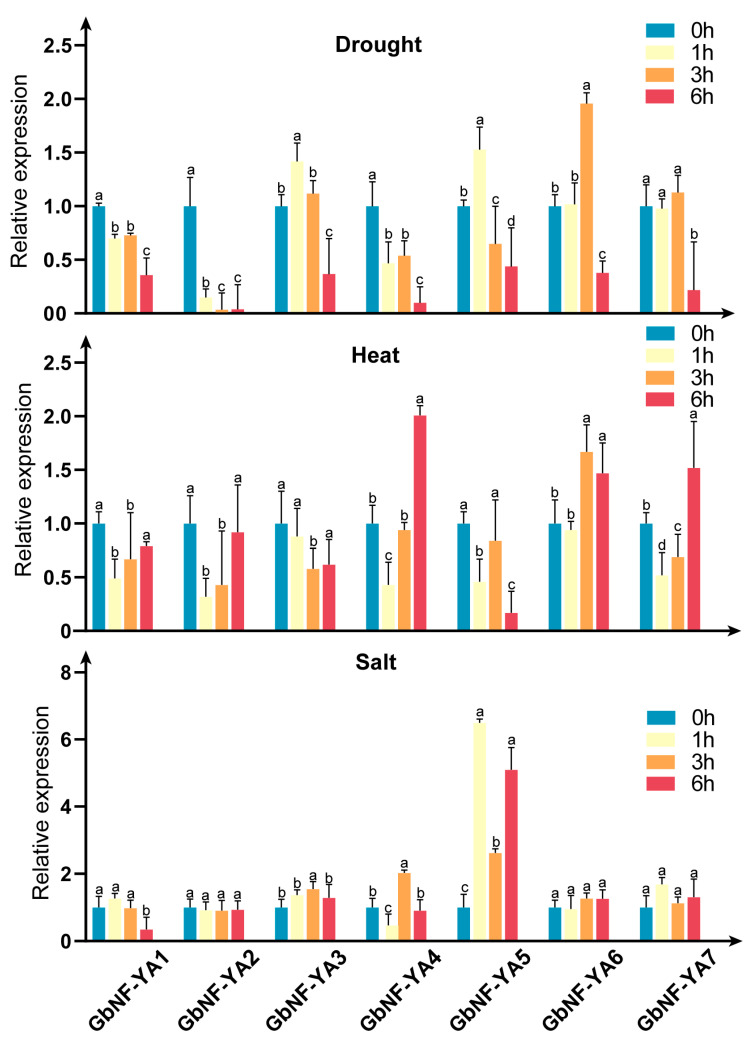
The expression profiles of seven *GbNF-YA* genes in 2 month old seedling *G. biloba* under various treatments including drought, salt, and heat. The relative expression (fold) of the *GbNF-YA*s in stress-treated plants (1, 3, and 6 h treatment) was compared to untreated control plants. The transcript levels were plotted, and the mean was calculated using three biological replicates (n = 3). Bars show standard deviation. Significance was determined using one-way ANOVA (*p* < 0.05), and letters were used to indicate any significant differences in each variant.

Analysis of the cis-acting elements revealed that the promoter of *GbNF-YA*s contains numerous abiotic stress-related elements, indicating possible involvement in abiotic stress responses in ginkgo plants. To examine this speculation, we performed qRT-PCR to analyze the expression levels of 7 *GbNF-YA* genes in ginkgo plants undergoing drought, salt, and heat treatments. The results are shown in Figure 7. Under heat treatment, the expression of *GbNF-YA6* was significantly upregulated at 3 h (least significant differences (LSD) test at 5% level, *p* < 0.001) and 6 h (LSD test at 5% level, *p* < 0.001). Moreover, the expression levels of *GbNF-YA4* and *GbNF-YA7* also increased at 6 h. Conversely, the expression of *GbNF-YA3* was downregulated under heat treatment. Notably, under drought treatment, the expression of *GbNF-YA6* was also significantly upregulated at 3 h (LSD test at 5% level, *p* < 0.001). In addition, the expression of both *GbNF-YA3* and *GbNF-YA5* increased at 1 h and then dramatically decreased at 6 h (LSD test at 5% level, *p* < 0.05) of drought treatment, whereas *GbNF-YA7* exhibited slight expression changes except at 6 h. Under the 200 mM NaCl treatment, compared with the control, the expression of *GbNF-YA5* was significantly upregulated within 6 h (LSD test at 5% level, *p* < 0.05), while other *GbNF-YA* genes did not show significant differences after salt stress.

### 2.7. Subcellular Localization and Functional Identification of GbNF-YA6 

**Figure 8:**
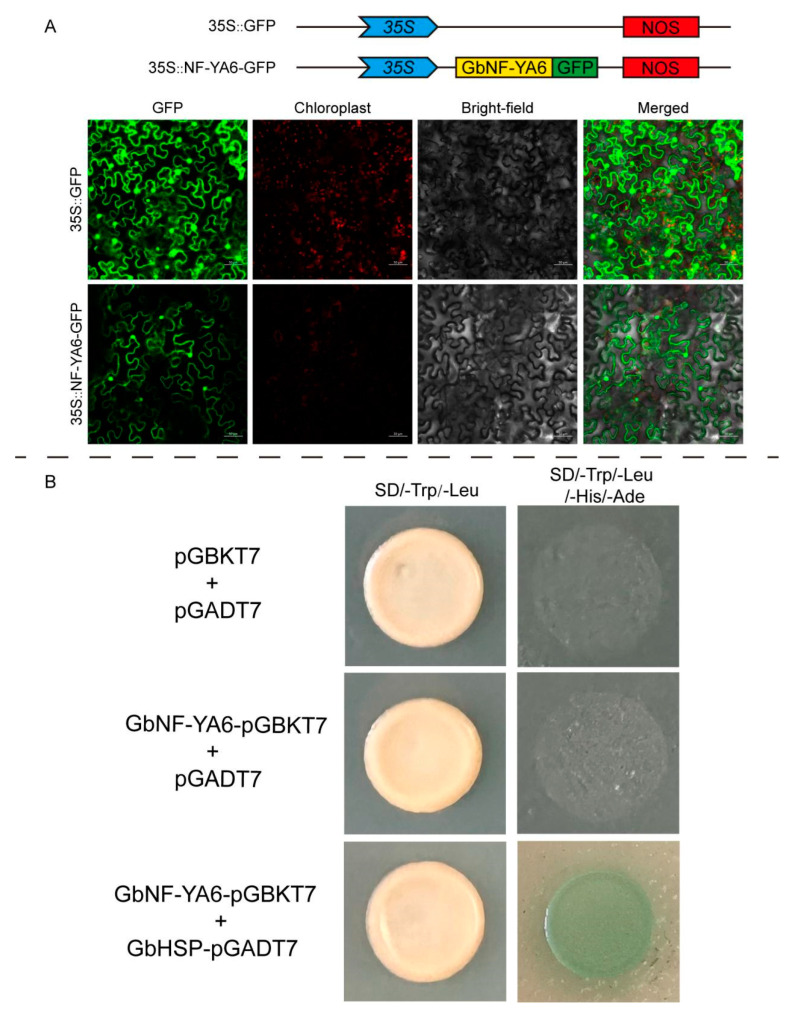
Subcellular localization and interacting protein of GbNF-YA6. (**A**) The subcellular localization of GbNF-YA6 was observed in the nucleus and membrane through confocal laser scanning microscopy. (**B**) The interacting protein of GbNF-YA6 was verified through the interaction between GbNF-YA6 and GbHSP in yeast by Y2H.

**Figure 9:**
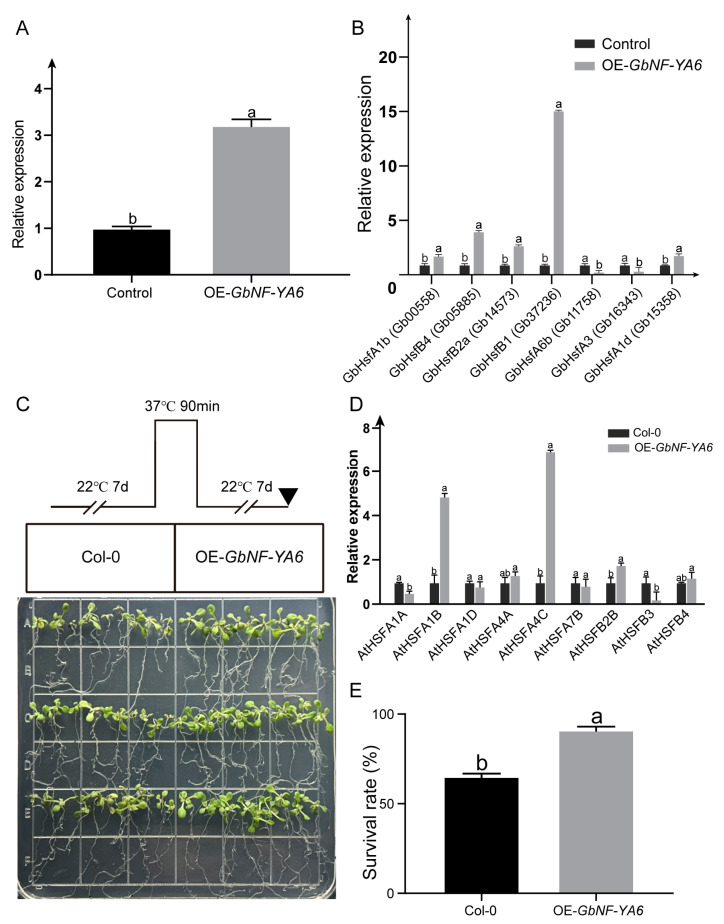
Functional characterization of *GbNF-YA6*. (**A**) The verification of overexpressed GbNF-YA6 ginkgo calli. (**B**) The expression of heat-shock genes in overexpressed *GbNF-YA6* ginkgo calli. (**C**) The growth state of Col-0 and overexpressed *GbNF-YA6* Arabidopsis under heat treatment. (**D**) The expression of heat-shock genes in overexpressed *GbNF-YA6* Arabidopsis. (**E**) The survival rate of Col-0 and overexpressing Arabidopsis *GbNF-YA6* under heat treatment. The transcript levels were plotted, and the mean was calculated using three biological replicates (n = 3). Bars show the standard deviation. Significance was determined using one-way ANOVA (*p* < 0.05), and letters are used to indicate any significant differences.

*GbNF-YA6* demonstrated significant changes in expression after exposure to both heat and drought treatments, as confirmed using qPT-PCR. This observation implies that *GbNF-YA6* may play a crucial role in providing resistance against these two forms of stress. Therefore, *GbNF-YA6* was selected for further study. The location of GbNF-YA6 was predicted in the nucleus. To confirm the prediction, we constructed 35S::GbNF-YA6-GFP and transformed it into *Nicotiana tabacum*. As shown in Figure 8A, *GbNF-YA6* was localized in the nucleus and membrane. To gain insight into the function of *GbNF-YA6*, we overexpressed it in ginkgo calli to evaluate the expression levels of heat-responsive genes, such as heat-shock transcription factors (HSFs), through qRT-PCR. The results in Figure 9A indicated that most HSF genes were upregulated in OE-*GbNF-YA6* ginkgo calli compared to the control. We also conducted the qPT-PCR of *AtHSF*s in 7 day old seedlings of overexpressed *GbNF-YA6* Arabidopsis and wild-type Arabidopsis. The result clearly showed that overexpressing *GbNF-YA6* in Arabidopsis significantly upregulated the expression level of *AtHSFA1B*, *AtHSFA4C*, and *AtHSFB2B*, whereas it downregulated *AtHSFA1A* and *AtHSFB3* (Figure 9B). Furthermore, three homozygous T3 transgenic Arabidopsis lines were identified and selected for thermotolerance analyses (Figure S1). The survival rate of three transgenic lines was significantly higher than that of the WT (Col-0) under heat stress conditions (Figure 9B), which indicated that the overexpression of *GbNF-YA6* can enhance the thermotolerance of transgenic Arabidopsis plants.

### 2.8. Protein Interaction of GbNF-YA6 

To clarify the mechanism of GbNF-YA6’s response to heat, we identified interactive proteins of GbNF-YA6 by screening a ginkgo protein library using a yeast two-hybrid system. The result showed that a HSP protein interacted with GbNF-YA6 (Figure 8B). We named it GbHSP (Gb_30137) (Figure S2). We further conducted Y2H assays to verify their interaction through the constructed GbHSP-pGADT7 and GbNF-YA6-pGBKT7 and yeast cell transformations. Yeast cells with GbNF-YA6 and GbHSP grew normally on selective medium (–T/–L/–H/–A) (Figure 8B), indicating a direct interaction between GbNF-YA6 and GbHSP. These results suggest that a regulatory module of GbNF-YA6 interaction with GbHSP may play an important role in response to heat stress in *G. biloba*, given the crucial role of HSP protein in heat resistance across many species.

## 3. Discussion

Gene duplication plays a crucial role in the expansion of gene families and can be a significant driver of evolutionary diversification [18]. Duplication events tend to result in increased functional diversity between duplicated gene pairs, and some new members may acquire novel functions or relinquish their original functions [19,20]. Duplication events also influence the evolutionary diversification of NF-Y family, resulting in a wide range of NF-Y member numbers among different plant species, such as Arabidopsis 36 NF-Y members (10 NF-YA, 13 NF-YB, and 13 NF-YC) [4], rice 34 NF-Y members (11 NF-YA, 11 NF-YB, and 12 NF-YC) [5], maize 50 NF-Y genes (14 ZmNF-YA, 18 ZmNF-YB, and 18 ZmNF-YC) [21], tomato 59 NF-Y genes (17 NF-YA, nine NF-YB, and seven NF-YC) [22], and soybean 68 NF-Y genes (21 GmNF-YA, 32 GmNF-YB, and 15 GmNF-YC) [6]. In woody plants, *P. trichocarpa* has 52 NF-Y genes (13 NF-YAs, 20 NF-YBs, and 19 NF-YCs) [23], while *Malus domestica* has 43 MdNF-Y genes (11 MdNF-YAs, 22 MdNF-YBs, and 10 MdNF-YCs) [24]. The NF-Y family is only reported in *Pinus tabuliformis* (28 members) and *Physcomitrella patens* (23 members) among gymnosperms and bryophytes [25,26]. In our study, we identified and confirmed a total of 25 NF-Y members (consisting of seven NF-YAs, 12 NF-YBs, and six NF-YCs) in the *G. biloba* genome. Although the genome size of gymnosperm species, such as *P. tabuliformis* (25.4 G) and *G. biloba* (9.87 G), is large, these two gymnosperm species have a relatively lower number of NF-Y family members compared to most reported angiosperm species. Furthermore, we did not find any tandem duplication events within the GbNF-Y family; instead, only four segment duplications were found within and among chromosomes. In contrast, more duplication events within the NF-Y family were found in many angiosperm species, such as maize and *P. trichocarpa* [21,23]. The lower number of identified NF-Y members in *G. biloba* may be explained by the fewer duplication events compared to angiosperm species.

However, since individual subunits of the NF-Y family cannot regulate transcription independently [12], they function in heterotrimers. The smaller number of GbNF-Y subunits does not necessarily mean a smaller function of the GbNF-Y family. In Arabidopsis, the 36 NF-Y subunits can theoretically form 1000 unique heterotrimeric combinations [27]. The gene diversity of each subunit of NF-Y contributes to the combinatorial diversity of the heterotrimeric complex. For example, most AtNF-YBs can interact with AtNF-YCs in Arabidopsis [28]. Comparing the protein sequences of GbNF-Ys and AtNF-Ys, we found that there is little difference in most conserved domains between Ginkgo and Arabidopsis, except for the DNA binding domain of GbNF-YAs and AtNF-YAs. This result indicated that, although the number of NF-Y family members varies among different species, the domains of the NF-Y family remain highly conserved. Therefore, NF-Y subunits in *G. biloba* might also form heterotrimeric complexes through interactions with each other, and a large number of trimeric transcription factor complexes could be involved in various biological functions. Moreover, to investigate the relationship of NF-Y proteins among Arabidopsis, Populus, and *G. biloba*, an unroot phylogenetic tree was constructed using complete NF-Y protein sequences. We found many orthologous genes (e.g., *GbNF-YA1*, *GbNF-YA3*, *GbNF-YA6*, *GbNF-YA7*, *AtNF-YA4*, *AtNF-YA7*, *PtNF-YA4*, *PtNF-YA7*, and *PtNF-YA9*) among these species, indicating that these genes share similar sequence characteristics.

Many transcription factors (including NF-Y) have been reported to be regulated by endogenous miRNAs [29]. In Arabidopsis, the NF-YA transcription factors have been identified to be targeted by four isoforms of miR169, which can regulate root architecture and facilitate nodule formation [30,31]. The interaction between miR169 and NF-YA transcription factor can also regulate the drought tolerance [32,33]. Moreover, in *Petunia hybrida*, 16 *PhNF-Y*s were reported to be the potential targets of 18 miRNA families showing varied expression patterns in different plant tissues [34]. In our study, we also found that *GbNF-Y*s were targeted by many endogenous miRNAs. For instance, *GbNF-YA2* and *GbNF-YA4* were targeted by 32 and 33 miRNAs respectively, while *GbNF-YC6* was targeted by only one miRNA. On the other hand, endogenous miRNAs also play significant roles in plant development, metabolism, and stress responses by targeting mRNAs for cleavage [35]. Previous studies have reported that miRNA169 can direct the cleavage of NF-YA mRNA to regulate abiotic stress responses. In *Brassica napus*, miR169n was confirmed to cleave the mRNA of *NF-YA8*, and overexpression of *NF-YA8* led to improved tolerance of drought stress [36]. Eight mature miR169s were shown to regulate seven *NF-YA* members in response to drought, ABA, and salt in *Zea mays* [37]. In our study, we found that miR169g and miR169m could cleave mRNA of *GbNF-YA4* according to previous degradome data [17]. These results suggest that targeted cleavage of NF-YA mRNA by miRNA169 in response to abiotic stresses might be conserved between gymnosperms and angiosperms.

The NF-Y family, constituting important transcription factors, plays vital roles in stress response [4]. In Arabidopsis, *AtNF-YA2*, *AtNF-YA3*, and *AtNF-YA5* were proven to participate in drought resistance [38]. In *Oryza sativa*, *OsNF-YA7* was also confirmed to improve drought tolerance in transgenic plants [9]. *PtNF-YA9* was reported to regulate resistance to drought stress in transgenic Arabidopsis [39]. *NF-YAs* were also found to participate in drought resistance in soybean [40]. Due to the cis-acting elements closely associated with gene function and regulatory mechanisms [41], we conducted an evaluation of the putative cis-regulatory elements in the 2000 bp putative promoter regions of all GbNF-Ys in the present study. The result showed that most *GbNF-Y*s contain various types of cis-elements related to plant growth and development (light responsiveness, circadian control, and seed-specific regulation), hormone response (auxin responsiveness, MeJA responsiveness, salicylic acid responsiveness, and gibberellin responsiveness), and stress response (drought inducibility, defense and stress responsiveness, and wound responsiveness). Recent study reported that NF-Y subunits could interact with MYB12 forming MYB12-NF-Y complexes, which can regulate flavonoid biosynthesis in tomato [42]. We also found flavonoid biosynthetic elements in the promoter regions of some GbNF-Ys members (*GbNF-YA2*, *GbNF-YC1*, *GbNF-YC5*, and *GbNF-YC6*), suggesting that these genes might also participate in the flavonoid synthesis. These analyses indicated that GbNF-Y members might also play important roles in growth, development, and stress response. qRT-PCR results showed significant differences in the expression levels of *GbNF-YA* members under heat, drought, and salt stress. Interestingly, we found that the expression of *GbNF-YA6* was significantly upregulated both under heat and drought stresses, indicating that *GbNF-YA6* might participate in the response to both heat and drought.

Heat stress is a major environmental stress that threatens plant metabolism and crop productivity [43,44]. Recently, it was discovered that *AtNF-YC10* interacts with *NF-YB3* through SUMOylation mediated by SIZ1 to improve heat tolerance in Arabidopsis [45]. However, only a few NF-YAs have been reported to be involved in heat stress. In our study, we found that transgenic *GbNF-YA6* Arabidopsis plants had a higher survival rate than the control under heat treatment, suggesting an important role in heat stress resistance. Heat-shock transcription factors (HSFs) are master regulators of the transcriptional regulatory network of plant heat stress, and they are strongly induced by heat stress [46]. In Arabidopsis, *HSFA1*s play a crucial role in quickly responding to heat stress. Additionally, other transcription factors including *HSFA2* and *DREB2A* are directly activated by *HSFA1*s [47,48]. Heat-shock proteins (HSPs) can also be rapidly induced by heat-shock transcription factors when plants are exposed to heat stress [49]. In the present study, transgenic ginkgo calli showed that the expression of some GbHSFs (e.g., *GbHSFB1* and *GbHSFB4*) can be induced by *GbNF-YA6*. Moreover, transgenic Arabidopsis showed upregulated expressions of *AtHSFA1B*, *AtHSFA4C*, and *AtHSFB2B* and downregulated expressions of *AtHSFA1A* and *AtHSFB3*. Y2H experiments verified that GbNF-YA6 can interact with GbHSP. Therefore, these results suggest that *GbNF-YA6* could enhance the heat tolerance of *G. biloba* by directly inducing *HSF*s expression or through interactions with HSP protein. However, further studies are warranted to investigate the mechanism by which *GbNF-YA6* regulates *HSF*s, contributing to heat tolerance.

## 4. Materials and Methods

### 4.1. Plant Materials

Two month old *G. biloba* seedlings were used for stress treatments in this study. They were grown in a climate chamber under 16 h of light (24 ± 1 °C) and 8 h of darkness (20 °C). For salt stress treatment, we used 200 mM NaCl, which is consistent with the treatment concentration described by Li et al. (2021) [50]. To induce drought and heat stress, we followed the previously described methods and treated seedlings with 20% PEG6000 and 40 °C, respectively [51]. Each treatment had three biological replicates. During the treatments, we harvested leaf samples at 0 h, 1 h, 3 h, and 6 h and immediately froze them with liquid nitrogen for future experiments. The frozen samples were stored at −80 °C until further use.

### 4.2. GbNF-Y Collection and Sequence Retrieval

The Ginkgo protein sequences were obtained by downloading them from the published genome [52]. To obtain the reference NF-Y protein sequences for *A. thaliana* and *Populus trichocarpa*, we accessed the Plant Transcription Factor Database (http://planttfdb.gao-lab.org/index.php accessed on 22 July 2023). These references were then used to search for similar protein sequences in the *G. biloba* genome using the BLAST program in TBtools, with E-values < 10^−5^. Next, we conducted a search for potential NF-Y domains in the entire genome via BLAST (Basic Local Alignment Search Tool) using the NF-Y domain as a reference. To ensure the presence of all major conserved domains in both the reference and the candidate sequences, candidates were confirmed using the NCBI (https://www.ncbi.nlm.nih.gov/Structure/bwrpsb/bwrpsb.cgi accessed on 22 July 2023) databases. Moreover, using DANMAN, we classified all *GbNF-Y* genes into *GbNF-YA*s, *GbNF-YB*s, and *GbNF-YC*s on the basis of their possession of specific conserved NF-Y and DNA-binding domains. Furthermore, we obtained information on each GbNF-Y gene including coding sequence (CDS), open reading frame (ORF) length, molecular weight (MW), and isoelectric point (PI), by searching ExPASy (https://web.expasy.org/protparam/ accessed on 22 July 2023).

### 4.3. Sequence Alignment and Phylogenetic Analysis

The protein sequence alignments of GbNF-Y were analyzed using MEGA 7.0 software. The served domains were marked using DNAMAN software (URL: http://www.bio-soft.net accessed on 22 July 2023). A phylogenetic tree was constructed using the maximum likelihood method with bootstrap values for 1000 replicates through IQ-TREE (v.1.6.9).

### 4.4. Analysis of the Gene Structure and Conserved Motifs of the GbNF-Y Family

To demonstrate the conserved motif structure of genes belonging to the GbNF-Y family, we submitted their protein sequences to the MEME motif search website (https://meme-suite.org/meme/ accessed on 22 July 2023). We set the parameters such that the maximum width was 300, and the maximum number of motifs was 20. Subsequently, we downloaded the results of the motif structure genes belonging to the GbNF-Y family and visualized them using TBtools. Alongside the analysis of conserved motifs, we also examined the gene structure of the GbNF-Y family. By utilizing the annotation of *GbNF-Y* genes and online analysis of their conserved motifs and domains, we scrutinized the gene structure of the GbNF-Y family and employed TBtools to visualize the resulting analysis.

### 4.5. Promoter Region Analysis of GbNF-Y Genes 

The promoter sequence of *G. biloba* genomic data was extracted using TBtools. The 2000 bp sequence before the translation initiation site of the gene sequence was preserved as the promoter sequence. cis-Acting elements were analyzed using Plant CARE online site (http://bioinformatics.psb.ugent.be/webtools/plantcare/html/ accessed on 22 July 2023). We normalized and adjusted the analysis results and visualized them through TBtools.

### 4.6. Genome Distribution and Gene Duplication of the GbNF-Y Family

The chromosomal distribution of *GbNF-Y* genes was illustrated using TBtools on the basis of their locations in the gff file. Furthermore, *GbNF-Y* genes with tandem or segmental duplications were identified with the help of MCScanX [53] and TBtools.

### 4.7. Prediction of miRNA Targeting the GbNF-Y Genes

The miRNA and degradome data of *G. biloba* were retrieved from our previously published datasets [17]. We sought all candidate *GbNF-Y* genes against available miRNA sequences using the psRNATarget Server (http://plantgrn.noble.org/psRNATarget/ accessed on 22 July 2023) and visualized potential networks of miRNAs and corresponding target genes using Cytoscape v3.5.1. The degradome data were used to identify the potentially cleaved targets of miRNAs.

### 4.8. RNA Extraction and Quantitative Real-Time PCR (qRT-PCR)

Total RNA was extracted from the leaves of all samples using the MiniBEST Plant RNA Extraction Kit (TaKaRa, Dalian, China) according to the manufacturer’s instructions. Next, cDNA was reverse-transcribed from the total RNA using the HiScript III RT SuperMix (Vazyme Biotech Co., Ltd., Nanjing, China). Lastly, the cDNA templates were utilized in further qRT-PCR experiments. The primers for the GbNF-YA genes were designed using Primer 5.0, and the GAPDH gene in *G. biloba* was employed as an internal reference. The primer sequences are listed in Table S1. The results of the qRT-PCR were analyzed using SPSS software for ANOVA.

### 4.9. Subcellular Location of GbNF-YA6

We cloned the sequence without terminator of *GbNF-YA6* and transformed it into the pRI-101 vector with a green fluorescent protein (GFP) tag, to create a fusion construct. The construct was then integrated into Agrobacterium GV3101 and introduced into tobacco leaves via injection, resulting in transient expression. Two days later, we observed the expression of GbNF-YA6-GFP using confocal laser scanning microscopy (LSM880; Carl Zeiss, Jena, Germany). As a control, we used pRI-101 expressing only GFP.

### 4.10. Yeast Two-Hybrid Assays

The pGADT7 and pGBKT7 vectors (Clontech) were utilized to conduct yeast two-hybrid (Y2H) assays. The pGADT7 vector consisted of a GAL4 activation domain, while the pGBKT7 vector contained a GAL4 binding domain. The complete open reading frame (ORF) of GbNF-YA6 was introduced into the pGBKT7 vector, whereas the ORF of GbHSP was inserted into pGADT7. The transformants were grown in synthetically defined medium with tryptophan and leucine (SD/-Trp/-Leu) and kept at 30 °C for 3 days. Subsequently, the transformants were transferred to synthetically defined medium with adenine, leucine, histidine, and tryptophan (SD/-Trp/-Leu/-His/-Ade) supplemented with X-α-GAL and incubated at 30 °C for 3 days. The empty pGADT7 and pGBKT7 vectors were employed as negative controls.

### 4.11. Ginkgo Callus Transformation

The coding sequences (CDSs) of *GbNF-YA6*, excluding the termination codons, were amplified from leaf cDNA to obtain full-length fragments. Subsequently, the amplified PCR fragments were incorporated into the expression vector pRI-101, driven by the cauliflower mosaic virus (CaMV) 35S promoter. For the transformation of ginkgo callus, recombinant plasmids were employed and transformed into 1 month old calli using the Agrobacterium tumefaciens GV3101-mediated approach. As a control, calli infected with Agrobacterium containing the empty vector (EV) were used. Supplementary Table S2 highlights the primers employed for the study. Additionally, the expression of heat-shock genes was conducted in both control and *GbNF-YA6*-overexpressing calluses.

### 4.12. Arabidopsis Transformation and Functional Assessment

The Agrobacterium tumefaciens GV3101 containing recombinant plasmids, which included the CDSs of *GbNF-YA6* without termination codons, was genetically transformed into Arabidopsis plants using the inflorescence infection method. The DNA of the infected Arabidopsis was then extracted to confirm the successful transformation of *GbNF-YA6* into the plants. Transgenic and Col-0 seeds were sown on the same MS medium; after incubation, they were placed in a culture room at 4 °C for 3 days. To compare the thermotolerance of the transgenic and wild-type (WT) plants, 1 week old seedlings were subjected to a 90 min treatment at 37 °C. After treatment, the plants were placed in the culture room for a 7 day recovery period, and the phenotypes and survival rates of the seedlings were recorded. The expression level of *AtHSF*s was also determined in transgenic Arabidopsis.

## 5. Conclusions

In this study, we identified 25 *GbNF-Y* genes present in the *G. biloba* genome. We characterized their chromosomal location, gene structure, physicochemical properties, protein structure, evolutionary relationships, and promoter cis-acting elements. A diverse range of stress response and growth-related cis-acting elements are present in the promoters of these genes. *GbNF-YA* genes can be induced by heat, or drought, or salt stress. The stress-related *GbNF-YA4* is targeted by miRNA169. Overexpressing *GbNF-YA6* in Arabidopsis improved the survival rate under heat and *GbNF-YA6* induced the expression of some HSFs and interacted with GbHSP, suggesting that *GbNF-YA6* improves heat tolerance by regulating HSFs or by interacting with HSP proteins. 

## Data Availability

Data sharing not applicable.

## Supplementary Materials

The following supporting information can be downloaded at https://www.mdpi.com/article/10.3390/ijms241512284/s1.
