# Supplementary material for: Genome-Wide Identification, Characterization, and Expression Analysis of NF-Y Gene Family in Ginkgo biloba Seedlings and GbNF-YA6 Involved in Heat-Stress Response and Tolerance
Source: Int J Mol Sci. 2023 Jul 31;24(15):12284. doi: 10.3390/ijms241512284 (PMC10418864; doi:10.3390/ijms241512284)
Supplement: Supplementary file 1 [file ijms-24-12284-s001.zip › Supplementary Figures and Tables.pdf]

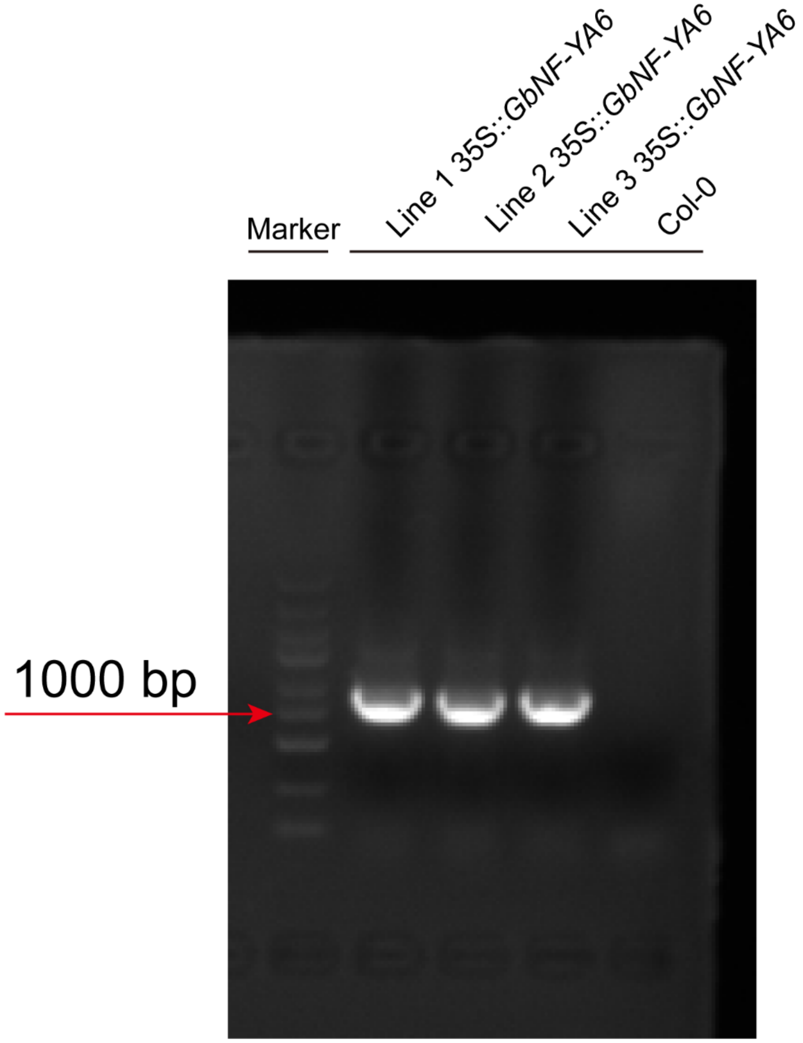

Figure S1. DNA validation of *GbNF-YA6* transgenic *Arabidopsis thaliana*.

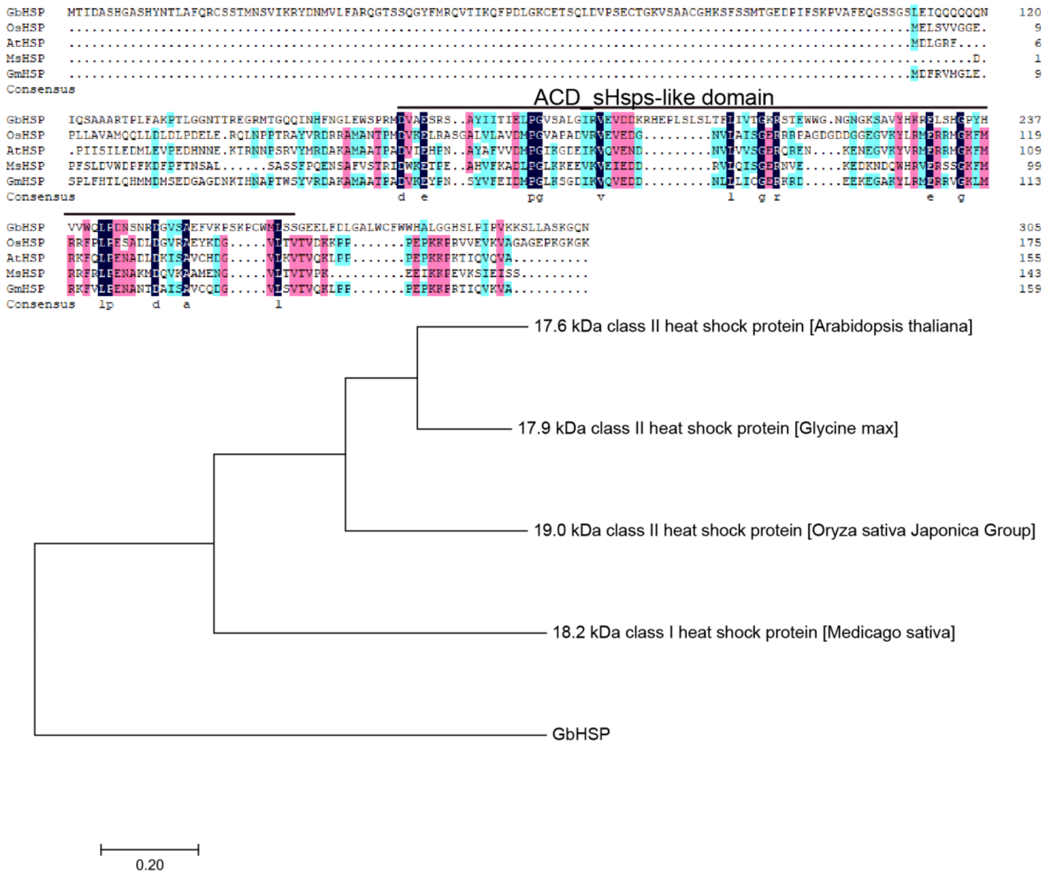

Figure S2. Analysis of phylogenetic tree and alignment of HSP genes among *Ginkgo biloba*, *Arabidopsis thaliana*, *Oryza sativa*, *Glycine max*, and *Medicago sativa*. The core, conserved domains of 5 HSP proteins were compared and visualized using DNAMAN. To construct phylogenetic trees, we used the neighbor-joining method implemented by MEGA software and calculated bootstrap scores from 1000 replicates.

Table S1. The ORF length, number of amino acids residues, calculated molecular weight, and isoelectric point of 25 GbNF-Y genes.

Name	Rename	Chr	ORF length (bp)	Number of amino acids residues (aa)	Calculated molecular weight	Isoelectric point (pI)
evm.model.chr1.645	GbNF-YA1	Chr1	985	322	36116.75	8.14
evm.model.chr2.1401	GbNF-YA2	Chr2	1241	406	45511.87	8.49
evm.model.chr5.832	GbNF-YA3	Chr5	634	207	23254.80	9.38
evm.model.chr5.1988	GbNF-YA4	Chr5	939	307	33669.43	7.07
evm.model.chr9.1443	GbNF-YA5	Chr9	765	250	27386.74	8.47
evm.model.chr10.208	GbNF-YA6	Chr10	820	268	29939.89	7.65
evm.model.chr11.1429	GbNF-YA7	Chr11	558	182	20213.15	9.61
evm.model.chr2.404	GbNF-YB1	Chr2	609	199	21478.86	6.07
evm.model.chr3.389	GbNF-YB2	Chr3	695	227	24662.19	7.2
evm.model.chr3.2277	GbNF-YB3	Chr3	710	232	25589.72	5.49
evm.model.chr5.1382	GbNF-YB4	Chr5	701	229	25158.76	5.63
evm.model.chr5.1791	GbNF-YB5	Chr5	701	229	25328.64	7.16
evm.model.chr6.1936	GbNF-YB6	Chr6	731	239	26120.76	6.66
evm.model.chr7.710	GbNF-YB7	Chr7	561	183	20150.54	5.35
evm.model.chr7.1924	GbNF-YB8	Chr7	518	169	18785.96	5.95
evm.model.chr8.688	GbNF-YB9	Chr8	545	178	20213.50	5.27
evm.model.chr11.521	GbNF-YB10	Chr11	695	227	24783.62	8.64
evm.model.chr11.1698	GbNF-YB11	Chr11	921	301	34560.48	6.40
evm.model.chr12.975	GbNF-YB12	Chr12	683	223	25125.12	8.85
evm.model.chr1.1773	GbNF-YC1	Chr1	844	276	31265.40	5.84
evm.model.chr3.676	GbNF-YC2	Chr3	820	268	29632.82	5.72
evm.model.chr5.1877	GbNF-YC3	Chr5	655	214	23974.20	6.12
evm.model.chr7.372	GbNF-YC4	Chr7	792	259	30120.50	6.23
evm.model.chr9.954	GbNF-YC5	Chr9	808	264	29713.69	7.77
evm.model.chr11.257	GbNF-YC6	Chr11	591	193	21953.24	6.08

Table S2. Primer sequences used in this study

Gene ID	Primer (5' – 3')
GbNF-YA1-qRT-PCR	F: CAGCAGCAGGTTCCGTCAACTG
	R: CGCCTCCAGAATGTCCTAGTTCAAG
GbNF-YA2-qRT-PCR	F: GCACAAGCGGGAAGTTCCTCAG
	R: CCTGACAACCCTGAAGCACCTTG
GbNF-YA3-qRT-PCR	F: GTGATGATAGCAGTGCAGCAGAGG
	R: TCAAGTTGAGTGTGTGGAGCCATG
GbNF-YA4-qRT-PCR	F: TCTCGCCATCGTCATGCAATGC
	R: ACCCTCTGATGCCTTTCCACTACC
GbNF-YA5-qRT-PCR	F: AATGATGGAAGCACAAGGAGGTTGG
	R: CAGTCACACCATGTATGAGCCACTC
GbNF-YA6-qRT-PCR	F: AACAACAGCAGCAGCAACAACAAC
	R: CATCACAAATTCCGCAGGAGGTAGG
GbNF-YA7-qRT-PCR	F: ACAACAACAGCCTCAGCATCCTAAG
	R: GAGCGTGCCACAATGTATCTCCTC
GAPDH	F: CTGCCAAGGCTGTAGGTAAGG
	R: TCAGATTCCTCCTTGATGGCG
Gb_00558-qRT-PCR	F: GACGACGCTTCCACAGATTC
	R: CAGGATCAACTTTCCTAAAACC
Gb_05885-qRT-PCR	F: TGTTTCTCCACAGCCTATG
	R: TTGAAGAATCGAGGTAGCA
Gb_14573-qRT-PCR	F: AAAGGAGGAAAGACAACCC
	R: GTTAGTGCTGCTCCATGAC
Gb_37236-qRT-PCR	F: AGGCTCATAGATCCATTCC
	R: GCTGGAGAAATTGTTATGC
Gb_11758-qRT-PCR	F: AAAGGAGGAAAGACAACCC
	R: GTTAGTGCTGCTCCATGAC
Gb_16343-qRT-PCR	F: TGTTTCTCCACAGCCTATG
	R: TTGAAGAATCGAGGTAGCA
Gb_15358-qRT-PCR	F: AAATCACCACCAGAAAAGG
	R: CACTATTGAGTCCGTCGCT
GbNF-YA6-pRI-101	F: gaccccgggggtaccggatccATGCAAGCTAGCCTTGATAGTAGTGC
	R: tttacccatgaattcggatccATCCGGACAAAGAACTGTGAAGA
GbNF-YA6-PGBKT7	F: aggccgaattcccggggatccATGCAAGCTAGCCTTGATAGTAGTGC
	R: ccgctgcaggtcgacggatccATCCGGACAAAGAACTGTGAAGA
GbHSP-PGADT7	F: gtgggcatcgatacgggatccATGACAATTGATGCTTCACATGG
	R: cagctcgagctcgatggatccGATGTTTTGGCCTTTAGATGCTAGC
ATHSFA1A-qRT-PCR	F: CGTTTCTTGACGGGTTCTCATCTCC
	R: GCCTGTGACTGTCCTGAAGTTATGG
AtHSFA1B-qRT-PCR	F: CATCATCCTCAAGCTGGTCTGGTTC
	R: ACTCTCCACTATCTGTCTCGCATCC

AtHSFA1D-qRT-PCR	F: GCACGAGCAAGCCAAAGCAATG
	R: GTCTCCATCTCTGTTCCCTCGGTAG
AtHSFA4A-qRT-PCR	F: CGATATGAACTGTGAGCCCGATGG
	R: TGCTACTGGAGGAGGAGGAGGAG
AtHSFA4C-qRT-PCR	F: CTCTCTTCCTCCAAGCAGTTCACAC
	R: AACCGCTCTTCTCGCATGATTCC
AtHSFA7B-qRT-PCR	F: GATATGGACGGCAACGGACTGATG
	R: TGCTTCACATTCGCCTCTTCTTCG
AtHSFB2B-qRT-PCR	F: GAAGTAGTGGATGTGGTGCTGGTG
	R: GGCGTCGGAATTGACCTCTGTG
AtHSFB3-qRT-PCR	F: CTTCCGCCACTTCATCCTCTTTCG
	R: TCCTCTGTATCTCTCCACCAACTCC
AtHSFB4-qRT-PCR	F: GCTCCGCCGCAGATTCCATTC
	R: GCCGCCGTGTCAATTTGTTGTG
Actin-qRT-PCR	F; TCACCACAACAGCAGAACGGGAAAT
	R: AAAGGACTTCTGGGCACCTGAATCTC
